# Breakfast Consumption and Academic Achievement Among Chinese Adolescents: A Moderated Mediation Model

**DOI:** 10.3389/fpsyg.2021.700989

**Published:** 2021-11-22

**Authors:** Chun Lei Gao, Nan Zhao, Ping Shu

**Affiliations:** School of Education, Jiangxi Normal University, Nanchang, China

**Keywords:** breakfast consumption, achievement motivation, socioeconomic status, academic achievement, moderating effect, mediating effect, Chinese adolescents

## Abstract

The studies have shown that a healthy lifestyle has a significant impact on the academic achievement of adolescents. Behavior of breakfast eating is considered a hallmark of dietary patterns and an important component of a healthy lifestyle. The prior study explained that students had a lower level of achievement motivation at school because they were exposed to some militating factors in their families such as absenteeism, ill health, malnutrition, and hunger. This study examined the mediating role of achievement motivation and moderating role of socioeconomic status (SES) in the association between breakfast consumption and academic achievement. This study used a sample of 15-year-old Chinese students who participated in Program for International Student Assessment (PISA) 2015. In terms of gender, female students accounted for 47.2% and male students accounted for 52.8%. The results showed that (1) breakfast consumption had a positive predictive effect on academic achievement; (2) achievement motivation played a partial mediating role in the relationship between breakfast consumption and academic achievement; and (3) the direct and indirect effects were moderated by the SES of students, which meant that the effect of breakfast consumption on achievement motivation can differ depending on the SES of students. Besides, both the effects were stronger for individuals with higher SES. The conclusion of this study has an important theoretical value and reference value to guide the Chinese parents and Chinese adolescents to pay more attention to breakfast consumption and healthy lifestyles.

## Introduction

Breakfast is the most important meal of the day, and the potential benefits of breakfast consumption for children, adolescents, and adults have been reported (Miller et al., 1998; Affenito, 2007; Timlin and Pereira, 2010). However, skipping breakfast is quite common among children and teenagers, and the phenomenon increases with age (Affenito et al., 2005; Rampersaud et al., 2005; Delva et al., 2006). This may be more common among certain minority ethnic or low socioeconomic groups and appears to be associated with other lifestyle factors that may be detrimental to health (Chang et al., 1994). Breakfast, as part of a healthy lifestyle, can have a positive impact on health and wellbeing of children (Julia et al., 2018). There is growing evidence that skipping breakfast or fasting has a negative impact on cognition, motivation, academic achievement, and test scores of children (Chang et al., 1994; Adolphus et al., 2019). Regular breakfast consumption is positively associated with academic achievement in children and adolescents (Adolphus et al., 2019). Hungry children may lack the energy and motivation to participate in classroom activities (Read, 1973), while malnutrition and micronutrient deficiency have been shown to affect physical, mental, and social health and reduce cognitive function (Leslie and Jamison, 1990; Scrimshaw, 1998; Worobey and Worobey, 1999). Behavioral research showed that skipping breakfast reduced the speed and accuracy of children with which children received information from memory (Pollitt, 1995). Little research has been carried out on the effects of breakfast eating behaviors, and little is known about the relationship between regular breakfast and academic achievement. In addition, earlier studies mainly focused on the frequency of breakfast, the factors affecting breakfast frequency and the impact of breakfast frequency on academic performance, while few studies explored the mediating and moderating mechanism of breakfast consumption.

While existing research on developing countries has found a fairly consistent trend – the members of the lower strata of society are living unhealthy lifestyles – much of the research in China does not support this view (Wang, 2019). In terms of diet, the nutrition transition theory showed that developing countries and low-income countries were experiencing the “Westernization” of the lifestyle, which was more obvious in China (Popkin, 2010). Wang et al. (2008) found that high-income Chinese families and urban residents purchase more snacks and fried foods than low-income families and rural residents. In other words, the lifestyles of high socioeconomic status (SES) groups are gradually shifting to unhealthy (Wang et al., 2008). In Hong Kong (China), people with secondary and tertiary education and jobs have less healthy lifestyles than those with less education and unemployment (Chan and Leung, 2015). Contrary to the earlier studies, the economy of China is still growing rapidly. The Chinese citizens are becoming more economically and educationally active and health conscious (Wang, 2019). At the same time, the Chinese students were reported to participate in mental and physical activities more frequently and at a more regular social pace (Julia et al., 2018). As the most important meal of the day, breakfast is an important symbol and component of a healthy lifestyle (Ruxton and Kirk, 1997; Reeves et al., 2013). The background of the SES changes the relationship between breakfast habits and academic achievement (Adolphus et al., 2019).

How and when does it affect the academic achievement of students? This study examined the effect of breakfast consumption on academic achievement using data from 15-year-old Chinese students participating in the Program for International Student Assessment (PISA) as a sample and further extends earlier research to examine the mediating role of achievement motivation and the moderating role of SES.

**FIGURE 1:**
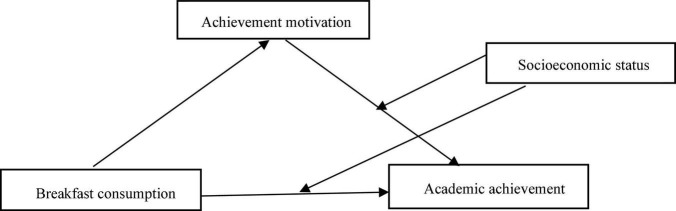
Conceptual model.

In summary, this study proposed a moderated mediating model (see Figure 1). The main objectives were twofold: (1) to examine whether achievement motivation mediates the relationship between breakfast consumption and academic achievement and (2) to examine whether SES moderates the mediating effect. The model deepens the direct relationship between breakfast consumption and academic achievement by answering not only “how” breakfast consumption affects academic achievement but also “when” breakfast consumption affects academic achievement (the hypothetical mediation diagram is shown in Figure 1).

### Achievement Motivation as a Mediator

Motivation is a process that corresponds to the intensity, direction, and persistence of efforts of an individual to achieve a goal (Robbins and Judge, 2013). Although the direct relation between the consumption of regular breakfast and achievement motivation has not been thoroughly explored in earlier studies, there are several reasons to support the above hypothesis. First, breakfast consumption contributes significantly to the nutritional adequacy of the overall diet (Julia et al., 2018). Adolescents who consume breakfast are more likely to have higher nutritional intake and a healthier and more adequate diet (Rampersaud, 2008). More importantly, there was a growing concern that poor health and nutrition of children may adversely affect their ability to learn in school, ultimately leading to low academic achievement (Pollitt, 1990; Chang et al., 1994; Nokes and Bundy, 1994). Skipping breakfast may lead to poor health and nutrition in children, which can affect the motivation of adolescents to achieve. Second, according to the Atkinson’s theory of expected value, initial motivation for high achievement comes from the influence of the family or cultural group in which the child lives, especially early education and training (Schultz, 1993). Studies have found that a supportive and caring relationship with parents positively predicts a greater interest in school, higher expectations for success, better self-regulation, and a perception of competence (Grolnick et al., 1991; Field et al., 1995; Jacobsen and Hofmann, 1997; Moss and St-Laurent, 2001; Wentzel, 2010). Previous studies have clearly shown that eating breakfast at home has a greater impact on intellectual performance than eating breakfast at school (Field et al., 1995). Breakfast consumption is associated with the health and wellbeing of children and adolescents (Julia et al., 2018). Therefore, making children feel the concern of their parents before school can also increase their achievement motivation. Third, previous research has shown that the more appealing the goal, the more subjective the motivation of an individual and the higher the achievement motivation (Schultz, 1993). We all know that the attractiveness of a goal is only the first step in achieving it, and persistence is also very important in achieving it. During this process, students need to maintain a high level of focus and attention. Spending sufficient time on task is an essential part of successful learning (Caroll, 1963). Previous studies have shown that students who skip breakfast feel hungry in class, resulting in poor concentration and difficulty paying attention in class (Pollitt, 1990; Nokes and Bundy, 1994); hungry children may lack the energy and motivation to become involved in classroom activities (Read, 1973). For adolescents consuming a small breakfast, provision of a mid-morning snack also resulted in less distraction from the task (Rampersaud, 2008). Thus, the decrease in achievement motivation may be due to the distraction caused by skipping breakfast.

Academic achievement and achievement motivation are considered to be the main indicators of learning process and outcomes of students (Islam and Chakrabarty, 2020). Academic achievement can be defined as “the knowledge and skills acquired in the disciplines of school.” Achievement motivation has been shown to significantly improve academic achievement (Lien, 2007; Islam and Chakrabarty, 2020). Achievement motivation can increase self-efficacy, attention, or focus, which are all related to perceptions of academic achievement (Lien, 2007). In summary, the hypothesis that breakfast consumption has a positive effect on achievement motivation is reasonable. Considering that breakfast consumption can improve achievement motivation and achievement motivation can also increase academic achievement, we put forward the following hypothesis:

**Hypothesis 1:** Achievement motivation would mediate the relation between breakfast consumption and academic achievement.

### Socioeconomic Status as a Moderator

Although breakfast consumption may improve achievement motivation and academic achievement, it is possible that not all adolescents are equally influenced. Therefore, it is important to examine the moderator that can influence the relation between breakfast consumption and its outcomes. Studies from several countries have shown that eating habits and dietary patterns of children are related to the SES of the family (Ahmed et al., 1998; Samuelson, 2000). Snacking and skipping breakfast are reported to be common among the Chinese teenagers (Huang et al., 2021). Previous research has shown that SES is a significant predictor of academic achievement and motivation of students (Phillips et al., 1998). The study showed that SES influenced the dietary style and academic achievement of adolescents in Hangzhou (Huang et al., 2021). Since adolescents from families with lower SES were more likely to have unhealthy eating behaviors, SES acted as an achievement buffer, which explained to some extent the relationship between dietary patterns and academic achievement (Florence et al., 2008; Hallström et al., 2012; Adolphus et al., 2013). SES background modified the association between habitual breakfast consumption and academic achievement (Adolphus et al., 2019). The study also found a significant interaction between SES and rare school-day consumption of breakfast. There was a link between SES and diet, as children and adolescents from families with higher SES may be more aware of healthy eating patterns (Adolphus et al., 2019). The present study examined whether the direct effect of breakfast consumption on academic achievement and whether the indirect effect of achievement motivation would be moderated by SES.

Traditionally, the PISA measure of SES is a weighted average of three indices, namely, parental educational attainment (in years), parental occupational status on the “International Socio-Economic Index” (ISEI) scale (Ganzeboom, 2010), and a measure of “household possessions.” The study supported that parental breakfast consumption, family monitoring, and family meal frequency were positively correlated with breakfast consumption or healthy food choice in children or adolescents (Keski-Rahkonen et al., 2003; Videon and Manning, 2003; Larson et al., 2007; Moore and Harré, 2007). Research has shown that family factors (e.g., education of father, education of mother, occupation of father, occupation of mother, family type, and monthly household income) could exert positive influences on breakfast consumption (Diaconu-Gherasim et al., 2020). Other studies have also pointed out that children of low SES are more likely to skip breakfast (O’Dea and Peter, 2001; Delva et al., 2006; Utter et al., 2007). However, members of upper class and urban population in China were the first to be exposed to the unhealthy elements of Western lifestyles (such as the consumption of calorie-dense, processed, and fried foods) (Wang, 2019). The continuous prominence of the Chinese tobacco and alcohol culture has also led to a healthier lifestyle for the upper class relative to the lower class (King and Ganotice, 2013). This Westernized lifestyle can influence the attitudes of parents toward eating breakfast. Therefore, do attitudes of the Chinese parents toward breakfast consumption have an impact on the breakfast behavior of adolescents?

The SES of family is a reflection of the social and economic resources that parents can provide (Ganzeboom et al., 1992; Bradley and Corwyn, 2002). Parental education and SES were found to be significant predictors of academic achievement and motivation of students (Phillips et al., 1998; Islam and Chakrabarty, 2020). The expectancy value theory has also proposed a mediational model in which perceived competence and achievement goals of children explain the relationship between family context (parental behaviors) with intrinsic motivation and academic achievement of students (Elliot, 2005; Wigfield et al., 2015). The relationship between academic achievement and achievement motivation was explored in terms of parental education, economic status, and occupation, respectively: first, numerous studies have shown a significant relationship between academic achievement of students and education of their parents (Kohl et al., 2000; Bakar et al., 2017). The importance of parental education in the academic success of students is an unquestionable assumption (Ogoye-Ndegwa, 2007). Another study showed that the higher the level of parental education, the higher the achievement motivation of the child (Acharya and Joshi, 2009), suggesting that the level of education directly influences the acquisition of achievement motivation in academic areas (Ogoye-Ndegwa, 2007). Second, according to a study of Pakistani school, there was a significant relation between their family income and their academic achievement in matriculation examination (Islam and Chakrabarty, 2020). In addition, it was found that the SES of parents was a significant predictor of academic motivation of students (Phillips et al., 1998; Muola, 2010). A previous study has shown that working parents can motivate their children to achieve better academic achievement and motivation than non-working parents (Muller, 1995). Above all, education level, economic status, and occupation of parents have significant effects on the academic achievement of adolescents as well as on the motivation.

According to earlier studies on the combination of mediation and moderation models (Muller et al., 2005; Edwards and Lambert, 2007; Hayes, 2013), there will be a moderated mediation model involving SES and achievement motivation in the relation between breakfast consumption and academic achievement. Thus, we put forward the following hypotheses:

**Hypothesis 2:** Socioeconomic status would moderate the relation between breakfast consumption and academic achievement and the relation being stronger in better socioeconomic conditions.

**Hypothesis 3:** Socioeconomic status would moderate the mediating effect of achievement motivation in the relation between breakfast consumption and academic achievement.

## Materials and Methods

### Samples

The Program for International Students Assessment is a massive international education evaluation program sponsored by the Organization for Economic Cooperation and Development (OECD). Beginning from 2000, PISA is administrated every 3 years to randomly selected groups of 15-year-old students in principally industrialized countries. Based on the reports of students to the contextual questionnaire, it helps to address relevant questions about educational opportunity and inequalities in learning outcomes (OECD, 2017a). PISA is held every 3 years, each time focusing on a field of assessment. The PISA 2015 survey focused on science, with reading, mathematics, and collaborative problem solving as minor areas of assessment (OECD, 2016). Thus, the scientific literacy of students was served as the academic achievement in this study.

Program for International Student Assessment adopts the two-stage sampling method (OECD, 2015). In the first stage, probability proportional to size (PPS) was adopted to extract schools. The larger the school, the larger the percentage. In the second stage, students were randomly selected from the selected samples, and every student in the same school who met the conditions of PISA test was selected with the same probability.

Approximately, 540,000 students completed the assessment in 2015, representing about 29 million 15-year olds in the schools of the 72 participating countries and economies (OECD, 2015). This study was conducted with a sample of the 15-year-old Chinese adolescents. Four of these entities, namely, Beijing, Shanghai, Jiangsu, and Guangdong, participated in PISA 2015; their combined results are reported as “B-S-J-G (China)” (OECD, 2016). In 2015, a total of 9,841 students completed the assessment, representing approximately 1.33 million 15-year-old students in “B-S-J-G (China)” schools (OECD, 2015). Missing data were deleted list-wise prior to analysis. After excluding missing data, the final sample included 8,000 15-year-old students (females = 3,774, males = 4,226) from 268 schools. The present work was carried out using data obtained in PISA 2015; therefore, the study sample was obtained from the above database.

### Variable Definitions

#### Breakfast Consumption

Program for International Student Assessment 2015 investigated the health behaviors of students in three main areas, namely, regular lifestyle, physical activities, and breakfast consumption (OECD, 2017a). The questionnaire data on whether students eat breakfast before school (YES = 2, NO = 1) were collected and analyzed.

#### Achievement Motivation

In 2015, PISA added the index of achievement motivation for the first time. PISA 2015 collected data about the achievement motivation of students using their responses to the five items (questions ST119Q01NA, ST119Q02NA, ST119Q03NA, ST119Q04NA, and ST119Q05NA in PISA 2015) measured on a four-point Likert scale (strongly agree, agree, disagree, and strongly disagree). Responses of “agree” or “strongly agree” were combined and are referred to agreement. An index, or overall scale, of achievement motivation was also derived from the responses of students, scaled so that higher scores on the index reflected stronger agreement to the items.

#### Socioeconomic Status

The PISA measure of SES has traditionally been built as a weighted average of three indices, namely, parental educational attainment (in years), parental occupational status on the ISEI scale (Ganzeboom, 2010), and a measure of “household possessions.” The PISA 2015 Technical Report (OECD, 2017b, pp. 339–340) describes ESCS as “a composite score derived *via* principal component analysis (PCA) from the indicators parental education, highest parental occupation, and household possessions including books in the home.”

In 2015, the “parental education” component of SCS was measured based on questions about the level of schooling of father and mother and the postsecondary educational qualifications of father and mother. The “parental occupation” component of SCS is measured based on open-ended questions about the job occupation of father and mother (questions ST014Q01TA and ST014Q02TA in PISA 2015). The “household possessions” component of SES was based on 25 items in questions ST011 (i.e., 16 dichotomous items, including three chosen by each country), ST012 (eight polytomous items, with a four-point scale), and ST013 (one polytomous item, with a six-point scale). The factor analysis was used to combine education level of parents, occupation level of parents, and family wealth status into the indicators of SES.

#### Academic Achievement

Traditionally, PISA assessed the outcome of students in terms of achievement tests. Because students complete different tests, science achievement cannot be obtained using traditional test scores but instead by using plausible values. Plausible values are multiple imputations of unobservable latent achievement for each student. Using item response theory, PISA 2015 used 10 plausible values to present each literacy achievement. These plausible values were calculated by averaging the scientific performance of all students taking the PISA test at 500 points with the standard deviation at 100 points (OECD, 2002). The dependent variable of this study was the first plausible value of scientific literacy of students.

### Procedure

In this study, we used the SPSS 23.0 software^1^ for the descriptive statistics and correlation analysis. Then, all variables were standardized into *Z*-scores using the SPSS 23.0 software, and the *Z*-scores of achievement motivation and science achievement were multiplied as the interaction term scores. The variance inflation factor of all the predictive variables was not higher than 1.20, so there was no multicollinearity problem. The moderated model and the moderated mediation model were tested using the SPSS macro process (Model 4 and Model 15) proposed by Hayes (2013). PISA 2015 data are public. Prior to data collection, the informed consent form and questionnaire have been reviewed and approved by OECD. Permission to conduct the study in the school was obtained from each principal and the informed consent of the students and their parents.

## Results

### Testing of Common Method Deviations

All variables in this study were measured by self-report of the subjects, which may lead to common method deviations. In order to reduce this possible deviation, Harman single factor test method (Podsakoff et al., 2003) was used to conduct exploratory factor analysis on all variables without rotation. The results showed that there were two factors with the characteristic root greater than 1, and the variation explained by the first factor is 39.4%, which was less than the critical value of 50%. Therefore, there was no obvious common method bias problem in this study. Besides, the study found that 7,473 students (93.41%) had breakfast before school, compared with 6.59% of students who skipped breakfast. The SES of the family students is normally distributed.

### Descriptive Statistics and Intercorrelations Between Variables

The correlation matrix of each study variable is shown in Table 1. Breakfast consumption and achievement motivation were significantly and positively correlated with academic achievement, indicating that they were all contributing factors to the academic achievement of adolescents. Moreover, SES was positively correlated with achievement motivation and academic achievement (the correlation between variables is shown in Table 1).

**TABLE 1:** Descriptive statistics and intercorrelations between variables.

Variables	*M*	SD	1	2	3	4
Gender	1.53	0.50				
1. Breakfast consumption	–	–	–			
2. Achievement motivation	0.392	0.763	0.148***	–		
3. Socioeconomic status	–0.842	1.114	0.029**	0.107***	–	
4. Academic achievement	531.388	97.457	0.050***	0.131***	0.293***	–

N = 8,000. **p < 0.01 and ***p < 0.001.

### Testing for the Proposed Model

First, Model 4 in SPSS macro compiled by Hayes (2013) (Model 4 is a simple mediation model) was used to test the mediating effect of achievement motivation on the relationship between breakfast consumption and academic achievement. The results (see Table 2) showed that breakfast consumption had a significant predictive effect on academic achievement (β = 0.791, *t* = 17.941, *p* < 0. 001), and the direct predictive effect of breakfast consumption on academic achievement was still significant (β = 0.186, *t* = 2.537, *p* < 0. 05) when the mediating variables were included. Achievement motivation had a significant effect on the prediction of academic achievement (β = 0.186, *t* = 16.568, *p* < 0. 001). In addition, the upper and lower limits of the 95% confidence intervals of the direct effect of breakfast consumption on academic achievement and the mediating effect of achievement motivation do not contain 0 (see Table 3), indicating that breakfast consumption can not only directly predict academic achievement but also predict academic achievement through the mediating effect of achievement motivation. The direct effect (0.115) and mediating effect (0.147) accounted for 43.89 and 56.11% of the total effect (0.262), respectively. Therefore, H1 was supported (the results of the mediation model are shown in Tables 2, 3).

**TABLE 2:** The Mediation Model Test of achievement motivation.

Outcome variable	Predictor variable	β	SE	*t*
Gender		–0.526	0.022	−2.388*
Academic achievement	Breakfast consumption	0.791	0.044	17.941***
Academic achievement	Breakfast consumption	0.186	0.011	2.537*
	Achievement motivation	0.186	0.112	16.568***

N = 8,000. Bootstrap sample size = 5,000. *p < 0.05 and ***p < 0.001.

**TABLE 3:** Total effect, direct effect, and mediating effect breakdown table.

	Effect	Boot SE	Boot LL CI	Boot UL CI	Relative effect value
Total effect	0.262	0.044	0.172	0.348	
Direct effect	0.115	0.045	0.026	0.203	43.89%
Indirect effect	0.147	0.011	0.125	0.170	56.11%

*LL, low limit; CI, confident interval; UL, upper limit.*

**FIGURE 2:**
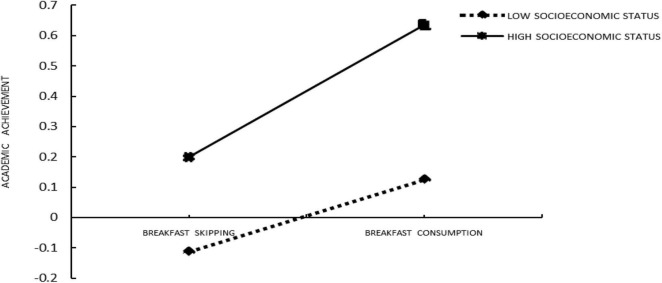
Socioeconomic status moderated the relation between breakfast consumption and academic achievement.

**FIGURE 3:**
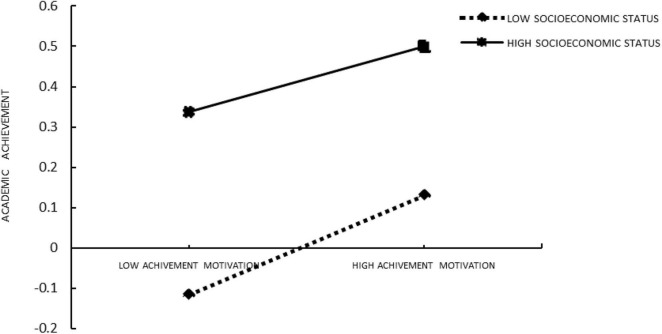
Socioeconomic status moderated the relation between achievement motivation and academic achievement.

Second, Model 15 in SPSS macros compiled by Hayes (2013) (Model 15 assumes that the latter half and direct path of the mediation model are adjusted, which is consistent with the theoretical model in this study) was used to test the moderated mediation model. The results (see Tables 4, 5) showed that the interaction of breakfast consumption and SES had significant predictive effects on academic achievement (β = 0.101, *t* = 2.557, *p* < 0. 05) so was the interaction between achievement motivation and SES (β = −0.042, *t* = −4.091, *p* < 0. 001). These findings indicated that the association between breakfast consumption and academic achievement and the association between achievement motivation and academic achievement were moderated by SES (see Figures 2, 3). Furthermore, as can be seen from the conditional direct effect analysis and conditional indirect effect analysis, two of the three conditional direct effects and all conditional indirect effects were positively and significantly different from zero. The study found that breakfast consumption had a significant effect on academic achievement when the SES was high. Achievement motivation of students was positively correlated with the SES. It can be seen from Figures 2, 3 that the relationship between breakfast consumption and academic achievement is stronger in students in families with high SES than students in families with low SES.

**TABLE 4:** Moderated mediation model testing.

Outcome variable	Predictor variable	β	SE	*t*
Gender		–0.051	0.02	−2.548*
Academic achievement	Breakfast consumption	0.118	0.041	2.868***
	Achievement motivation	0.126	0.010	12.197***
	Socioeconomic status	0.411	0.010	40.710***
	Breakfast consumption × socioeconomic status	0.101	0.040	2.557*
	Achievement motivation × socioeconomic status	–0.042	0.010	−4.091***

N = 8,000. Bootstrap sample size = 5,000. *p < 0.05 and ***p < 0.001.

**TABLE 5:** The direct and mediating effects on the different levels of socioeconomic status.

	β	Boot SE	Boot LL CI	Boot UL CI
**Conditional direct effect analysis at IA = *M* ± SD**				
M−1 SD (−1.0)	0.017	0.054	−0.089	0.123
*M* (0)	0.118	0.041	0.038	0.199
*M* + 1 SD (1.0)	0.220	0.060	0.102	0.338
**Conditional indirect effect analysis at IA = *M* ± SD**				
*M*−1 SD (−1.0)	0.133	0.014	0.108	0.161
*M* (0)	0.099	0.010	0.082	0.119
*M* + 1 SD (1.0)	0.067	0.011	0.044	0.089

*LL, low limit; CI, confident interval; UL, upper limit.*

The findings suggested that SES played a moderating role not only in the direct prediction of breakfast consumption on academic achievement but also in the predictive role of achievement motivation on academic achievement. Therefore, both H2 and H3 were supported (the results of moderated mediation are shown in Tables 4, 5, the effect results of moderating variables are shown in Figures 2, 3).

## Discussion

In this study, a moderated mediation model was constructed to analyze the relationship between breakfast consumption and academic achievement in adolescents. The results indicated that achievement motivation played a mediating role, and the SES played a moderating role in the relation between breakfast consumption and academic achievement. Besides, the mediating effect of achievement motivation was also moderated by SES. First, our study found that breakfast consumption could significantly predict academic achievement in adolescents. This result was consistent with previous study (Lozano and Ballesteros, 2006). Students who do not eat breakfast will lose their concentration and motivation in class because of hunger, which will greatly reduce the effective learning time in class (Read, 1973; Chang et al., 1994). Students who are in such a state for a long time will inevitably lead to a decline in their academic achievement. In addition, skipping breakfast for long periods of time can lead to malnutrition, which can lead to a decline in the learning performance of students, as has been shown in previous studies (Pollitt, 1995; Scrimshaw, 1998; Worobey and Worobey, 1999). All of these can influence the academic achievement of adolescents. Second, our study indicated that achievement motivation partially mediated the relation between breakfast consumption and academic achievement. It has been shown that achievement motivation is a positive factor influencing academic achievement (Lien, 2007). Consistent with our hypothesis, this study found a positive effect of breakfast consumption on achievement motivation, and achievement motivation partially mediated the effect of breakfast consumption on academic achievement. Previous research has explained that students were less motivated to achieve in school because they were exposed to a number of disadvantages at home, such as absenteeism, poor health, malnutrition, and hunger (Muola, 2010). Eating breakfast can alleviate hunger, improve concentration of students in class, and increase motivation of students to learn by making them feel cared for by their parents. These can further influence the achievement motivation of adolescents.

In addition, the study found that the education and SES of parents were the significant predictors of academic achievement and motivation of students (Winter et al., 1998). Our study found that the effect of breakfast consumption on academic achievement, as well as the effect of breakfast consumption on academic achievement through achievement motivation, was moderated by SES. As an old Chinese saying goes, “A child from a poor family takes charge early.” Due to the relative poverty of the living environment, when students from poor families have similar learning motivation as students from rich families, their economic situation has been improved in the same way. Poor students may want to change their current situation more urgently and with stronger motivation than those students who are better (Chen et al., 2018). They may believe that their economic situation has improved and that they are more likely to achieve excellence if they study harder. SES not only prompted the direct impact of breakfast consumption on academic achievement but also influenced the indirect impact of achievement motivation on academic achievement.

Specifically, the moderating effect of SES between breakfast consumption and its outcomes (e.g., achievement motivation on academic achievement) may be explained by the following reasons. Students with high SES imply that their parents have a higher level of educational level, economic status, and professional status. First, the study showed that parents with higher education level made breakfast more frequently and more efficiently than parents with lower education level (Muola, 2010), ensuring both nutritional intake for students and a sense of family love for their children. Second, the occupation of parents influenced the inclination of students to choose career, which motivated them to get better grades (Muller, 1995). Third, students who live in better-quality houses, with better facilities and more learning materials, and at least three meals/day have better achievements in their schools (Ngware et al., 2007). Although members of the upper class are most influenced by Western lifestyle (King and Ganotice, 2013), higher affordable Chinese residents consume higher priced but unhealthy food (King and Ganotice, 2013; Wang, 2019). Shi et al. (2005) reported the results of a cross-sectional study on the relationship between dietary habits, food preferences, and socio-demographic factors among 12- to 14-year-old adolescents in Jiangsu Province, China. The results showed that most Chinese teenagers usually eat three meals a day (Shi et al., 2005). This proved that the Chinese families attach great importance to breakfast. More interestingly, we found in this study that the higher the economic status of the family, the higher the level of concern about the breakfast consumption behavior of their children.

## Limitations

There were some shortcomings in this study, which needed to be improved in future studies. First, this study used a cross-sectional research design, which prevented the results from being extrapolated to a causal relationship. Future studies should use a longitudinal design or experimental approach to investigate the causal relationship between breakfast consumption and academic achievement. Second, since the 2015 PISA data mainly tested the science literacy of 15-year-old students, this study mainly used science literacy scores as a variable for the academic achievement of students, and future studies will provide a comprehensive analysis of student achievement. Third, this study did not investigate the number of students eating breakfast every week, or the types of breakfasts students eat, which is the biggest limitation of this study. Future studies need to investigate this further.

## Implications

This study had significant practical implications. First, it was necessary for parents and educators to help adolescents know that breakfast consumption had positive impacts on academic achievement and to help them develop the habit of eating breakfast. Second, considering that achievement motivation is a vital mechanism linking breakfast consumption and academic achievement, it would be effective for parents and educators to help adolescents improve their grades. Eating breakfast can further improve the academic achievement of students by reducing hunger and improving concentration to increase motivation. Third, SES influenced not only the direct effect of breakfast consumption on academic achievement but also the indirect effect of breakfast consumption on academic achievement through the mediation of achievement motivation. Therefore, by analyzing the results, we can make the following suggestions: (1) families with lower SES can reduce the impact of financial problems on academic achievement and motivation of children by providing them with breakfast regularly every day; (2) parents can increase the importance of breakfast and cultivate good eating habits in their children; and (3) the government should increase compensatory investment and provide resource support for disadvantaged groups.

## Conclusion

China is currently in a phase of rapid development and transition, and the dietary patterns of residents have changed significantly. This study was the first attempt to examine the mediating role of achievement motivation between breakfast consumption and academic achievement and the moderating role of SES among the Chinese adolescents. It deepened prior studies by examining the mechanisms underlying this relationship. Specifically, it explained how, when, and when of how breakfast consumption influenced academic achievement.

Breakfast consumption was positively correlated with achievement motivation and academic achievement of students, and the SES of family also played a good moderating role. The results of this study suggested that breakfast was necessary to promote healthy eating among the Chinese adolescents and their parents. The preservation of traditional Chinese eating habits should be a common concern for parents and schools. The government should provide breakfast guarantee for students from disadvantaged families. Further research is needed to investigate the possible ways in which SES influences eating habits.

## Data Availability Statement

This study was carried out using data obtained in PISA 2015; therefore, the study sample was obtained from the above database, available at: https://www.oecd.org/pisa/data/2015database/.

## Ethics Statement

PISA 2015 data are public. Permission to conduct the study in the school was obtained from each principal and the informed consent of the students and their parents.

## Author Contributions

CG was responsible for the topic selection and data integration of the manuscript. NZ was responsible for the data analysis, method selection, and manuscript writing and revision. PS was responsible for the data analysis. All authors contributed to the article and approved the submitted version.

## Conflict of Interest

The authors declare that the research was conducted in the absence of any commercial or financial relationships that could be construed as a potential conflict of interest.

## Publisher’s Note

All claims expressed in this article are solely those of the authors and do not necessarily represent those of their affiliated organizations, or those of the publisher, the editors and the reviewers. Any product that may be evaluated in this article, or claim that may be made by its manufacturer, is not guaranteed or endorsed by the publisher.

## Footnotes

1
https://www.ibm.com/cn-zh/analytics/spss-statistics-software
